# Measurement of Mass Flow Rates of Petrochemical Particles Based on an Electrostatic Coupled Capacitance Sensor

**DOI:** 10.3390/s25226850

**Published:** 2025-11-09

**Authors:** Yipeng Li, He Meng, Guangzu Wang, Jian Li

**Affiliations:** 1 SINOPEC Research Institute of Safety Engineering Co., Ltd., Qingdao 266000, China; 2 National Engineering Research Center of Power Generation Control and Safety, School of Energy and Environment, Southeast University, Nanjing 211189, China

**Keywords:** electrostatic coupled capacitance, measurement system, petrochemical particle, mass flow, velocity, concentration

## Abstract

To enable real-time monitoring of particle mass flow rate in petrochemical pneumatic conveying systems, thereby facilitating process control optimization and improving energy efficiency, an online measurement system for petrochemical particle mass flow based on a non-intrusive electrostatic coupled capacitance sensor is developed. The measurement system determines particle flow velocity by analyzing electrostatic signals using a cross-correlation method, and calculates particle concentration by applying a pre-calibration that correlates capacitance signals with concentration values. These two parameters are then combined to calculate the real-time particle mass flow rate. The performance of the developed system is evaluated under different pipe diameters and particle concentration ranges, in both lab-scale and pilot-scale pneumatic conveying rigs. The obtained results show that the measurement system achieved a maximum relative error of 5.5% for mass flow measurements in the lab-scale 50 mm pneumatic conveying pipeline when the particle concentration range was between 2.04 kg/m^3^ and 6.43 kg/m^3^. As for the pilot-scale 100 mm pneumatic conveying, the maximum relative error of the particle concentration measurement was 3.6% when the particle concentration range was 30.98~68.87 kg/m^3^. These results demonstrate that the developed system has strong adaptability and reliability, highlighting its broad potential for industrial applications.

## 1. Introduction

Petrochemical particle materials, such as polyethylene and polypropylene, are essential raw materials in modern industry [1,2,3]. During processes such as particle granulation, drying, and packaging, petrochemical particles are typically transported through pipelines in the form of gas-solid two-phase flow. Real-time monitoring of their mass flows not only facilitates process control optimization [4], but also plays crucial roles in stabilizing product quality, improving energy efficiency, and reducing equipment wear.

Various measurement methods have been developed for gas-solid two-phase flow parameters, including electrical, optical, acoustic, and microwave techniques [5,6,7,8,9,10]. Among these, electrical methods have attracted significant attention due to their non-intrusive nature, simple structure, low cost, high reliability, and non-contact measurement capability. Electrical methods primarily include electrostatic and capacitance sensing. Electrostatic sensing detects charged particles based on the principle of electrostatic induction, and is often combined with cross-correlation algorithm or spatial filtering technique to calculate particle flow velocity [11,12]. Capacitance sensing, on the other hand, obtains particle concentration information by detecting changes in the dielectric constant within the measurement region [13]. Encouraging studies have combined the two to achieve complementary information and enhance measurement capabilities. Li et al. developed an integrated electrostatic and capacitance sensor system for online measurement of particle velocity, concentration, and mass flow in dense-phase pneumatic coal conveying [14]. Electrostatic sensing can also be combined with electrical capacitance tomography (ECT) for measurement of particle parameters and investigation of flow characteristics in particulate processes [15,16]. It is obvious that the integration of electrostatic and capacitance sensors potentially offers more sensing information, enabling estimation of more flow parameters. However, simple sensor integration is usually incompact, and cannot acquire the flow information within the same sensing space, and this may reduce measurement precision. Considering the structural and mathematical similarities between electrostatic and capacitance sensors, an electrostatic coupled capacitance sensor was proposed in [17] to simultaneously acquire electrostatic and capacitance signals within the same sensing space, potentially offering a simplified system structure and better measurement precision. Further, in [18], a non-contact ring-shaped electrostatic coupled capacitance sensor was developed to effectively improve the reliability of particle velocity and concentration measurements [18]. Although electrostatic coupled capacitance sensors have made progress in particle parameter measurement, systematic validation of sensor adaptability, accuracy, and stability under practical operating conditions remains limited. Furthermore, lab-scale validations are typically restricted to fixed pipe diameters, limited particle concentration ranges, and limited operational conditions, constraining their application in complex industrial environments. An instrument system should also be well designed for long-term working in an industrial environment.

In this study, an online measurement system for petrochemical particle mass flow based on an electrostatic coupled capacitance sensor is developed. The system’s performance is evaluated under different pipe diameters and particle concentration ranges in both laboratory- and pilot-scale pneumatic conveying rigs. The measurement results are then analyzed and presented.

## 2. Measurement System Development

### 2.1. Measurement System Design

**Figure 1:**
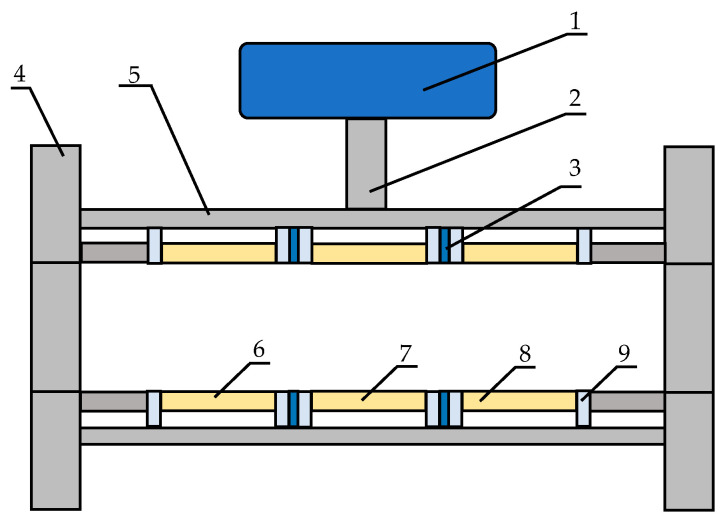
Schematic diagram of an integrated measurement system for an electrostatic coupled capacitance sensor. 1—circuit box; 2—connecting boss; 3—isolation electrode; 4—flange; 5—shielding cover; 6—upstream detection electrode; 7—excitation electrode; 8—downstream detection electrode; 9—insulating ring.

A schematic of the electrostatic coupled capacitance sensor system developed in this study is shown in Figure 1. The system mainly consists of a sensing unit and a signal-conditioning circuit. The sensing unit comprises an excitation electrode, upstream and downstream detection electrodes, isolation electrodes, insulating rings, a flange, and a shielding cover. The cover and all electrodes are made of stainless steel. The key geometrical parameters of the sensor are denoted as follows: inner diameter (*D*); axial length of the excitation and detection electrodes (*W*_1_); axial length of the isolation electrode (*W*_2_); and axial length of the insulating ring (*W*_3_). The signal-conditioning circuit is housed in a separate enclosure and connected to the sensing unit through a dedicated mounting boss. The signals are acquired using a digital signal processor for calculation of particle flow parameters.

**Figure 2:**
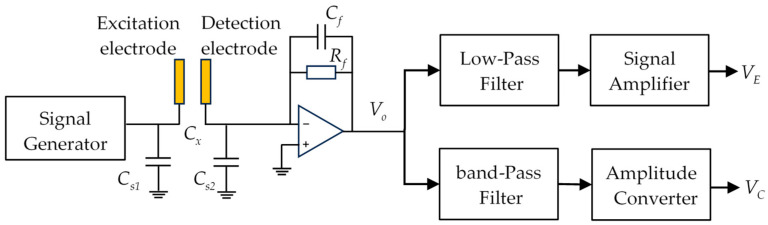
Schematic diagram of signal detection circuit.

According to the fundamental principle of electrostatic coupled capacitance sensors [17], the raw output signal contains both electrostatic and capacitance components. These two components exhibit markedly different frequency characteristics: the electrostatic signal generally lies below 1 kHz, and is primarily determined by the particle velocity and the sensor width, whereas the capacitance signal is governed by the high-frequency excitation voltage applied to the excitation electrode, and typically lies in the megahertz (MHz) range. Exploiting this frequency separation, the filter modules in the signal-conditioning circuit (Figure 2) can effectively isolate and extract the two signals independently. Placing the excitation electrode between the upstream and downstream detection electrodes results in two similar electrostatic signals and two similar capacitance signals being obtained simultaneously. The particle velocity can be calculated by correlating either the electrostatic signals or the capacitance signals, giving the sensing system a wider adaptability to flow variations.

### 2.2. Particle Parameter Calculation

In this study, the mass flow rate of particles in a pneumatic conveying process is not measured directly but is determined by separately measuring particle velocity and concentration and then computing their product. Typically, the particle velocity can be obtained by applying a cross-correlation algorithm to the electrostatic or capacitance signals.

When solid particles pass through the sensing region, the signals from the two detection electrodes exhibit a transit time *τ*_0_ in the time domain. This *τ*_0_ corresponds to the time lag at which the cross-correlation function *R_xy_*(*τ*) of the two signals reaches its maximum, as given by Equation (1):

(1) $$R_{xy}\left(\tau \right)\;=\;\frac{1}{T}\int_{0}^{T}x\left(t\right)y\left(t-\tau \right)\mathrm{dt}$$

where *x*(*t*) and *y*(*t*) are the signals from the upstream and downstream electrodes, *T* is the length of the time window for analysis, and *τ* is the time delay variable. The average particle velocity *v* through the sensor can then be expressed as

(2) $$v=L/\tau_{0}$$

where *L* is the axial spacing between the centers of the upstream and downstream detection electrodes, and *τ*_0_ is the time delay.

The capacitance signal is used to measure particle concentration. During measurement, a voltage is applied to the excitation electrode, and the detection electrode produces a capacitance signal *C_x_* that increases monotonically with particle concentration (*β*). The variation in capacitance Δ*C_x_* is converted by the measurement circuit into a corresponding change in voltage Δ*V_C_*. The relationship between Δ*V_C_* and *β* can be expressed as

(3) $$\;\beta =f\left(\Delta V_{C}\right)$$

Once particle velocity and concentration are obtained, the mass flow rate *M* of solid particles across the cross-section of the pneumatic conveying pipe can be calculated as

(4) M=A·v·β

where *A* is the cross-sectional area of the sensing device. For measurement of mass flow rates of solid particles, the measurement uncertainty depends on the measurement uncertainties of velocity and concentration. The measurement system should be carefully designed to obtain a satisfactory level of measurement accuracy.

## 3. Experimental Setups

Two types of laboratory tests were conducted: a belt–pulley experiment and a pneumatic conveying rig with particles. The belt–pulley setup was used to validate the measurement system in terms of velocity measurement accuracy and stability, whereas the lab-scale pneumatic conveying rig assessed the overall performance in measuring real particle-flow parameters (velocity, concentration, and mass flow rate) of polypropylene pellets. The sensor used in the lab-scale tests had the following dimensions: *D* = 50 mm, *W*_1_ = 40 mm, *W*_2_ = 10 mm, and *W*_3_ = 5 mm. To further evaluate the proposed measurement system, it was installed on a pilot-scale rig for pneumatic conveying of polypropylene pellets. The dimensions of the sensor in the pilot-scale tests were *D* = 100 mm, *W*_1_ = 40 mm, *W*_2_ = 10 mm, and *W*_3_ = 5 mm. Polypropylene pellets with a density of 0.90 g/cm^3^ and a mean particle diameter of 3.85 mm were used in both the lab-scale and pilot-scale pneumatic conveying experiments. For both the lab-scale and pilot-scale measurement sensors, the correlation distances of the electrostatic signals and the capacitance signals were 120 mm and 60 mm, respectively. The uncertainty of the correlation distance was 0.5 mm, which was mainly assembly error and could be easily controlled. In all experiments, the signals were acquired at a sampling frequency of 20 kHz. The uncertainties of time delay were 0.83% and 1.67% for the electrostatic and capacitance signals. Thus, a simple maximum search was used to determine the time delay with sufficient accuracy.

### 3.1. The Belt–Pulley Setup

**Figure 3:**
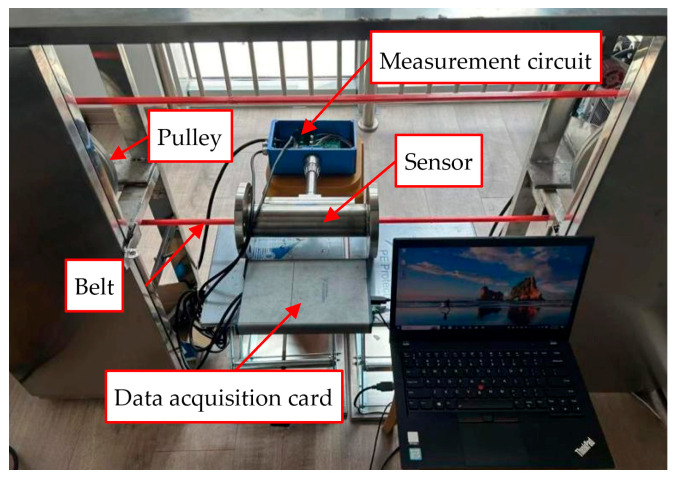
The belt–pulley setup.

The belt–pulley setup (Figure 3) consists of a motor-driven closed-loop polyurethane belt with a trapezoidal cross-section running at an adjustable and stable speed. Friction between the belt and the drive pulley causes electrostatic charge accumulation on the belt surface, effectively mimicking the charging of particle flow. The belt–pulley setup is used to validate the system in terms of velocity measurement accuracy and stability. Given that the actual velocity of the belt (*v*_r_) exhibits a stable linear correlation with the motor speed (*r*_m_), and considering that rm can be precisely regulated, the value of *v*_r_ is thus ascertainable. Thus, comparing the measured velocity of the belt (*v*_c_) using the measurement system with *v*_r_ enables evaluation of the accuracy and stability of the velocity measurement.

### 3.2. The Laboratory-Scale Pneumatic Conveying Rig

**Figure 4:**
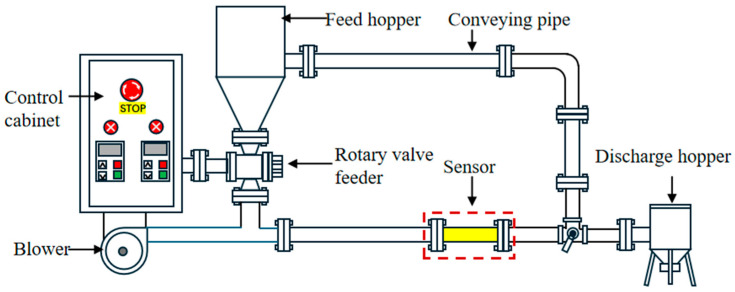
Schematic diagram of the laboratory-scale pneumatic conveying rig for particles.

The lab-scale pneumatic conveying rig for particles (Figure 4) comprises a feed hopper, a rotary valve feeder, a blower, a control cabinet, conveying pipes, and a discharge hopper. The pipe inner diameter is approximately 50 mm, and the electrostatic coupled capacitance sensor is installed in the test section of the conveying line. Polypropylene pellets are fed from the hopper. The mass feed rate is controlled by adjusting the rotary valve frequency, while the airflow rate is controlled by the blower frequency. The control cabinet provides unified adjustment of both the blower frequency and the rotary valve frequency. A weighing device is placed under the discharge hopper to obtain the cumulative mass of pellets conveyed over a given time by an offline gravimetric method.

### 3.3. The Pilot-Scale Pneumatic Conveying Rig

**Figure 5:**
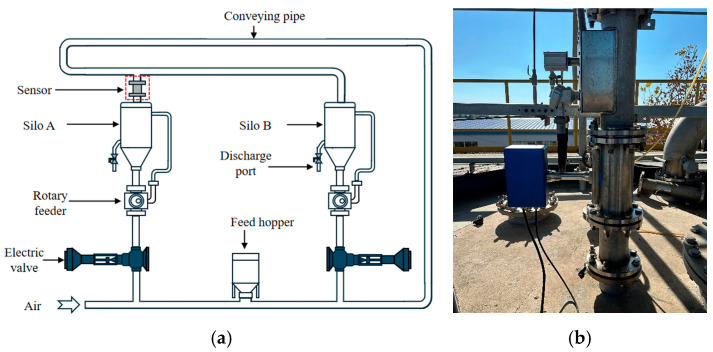
Schematic diagram of the pilot-scale rig for pneumatic conveying of polypropylene pellets: (**a**) Device structure; (**b**) sensor installation position.

Figure 5a illustrates the layout of the pilot-scale pneumatic conveying rig. The silo has a diameter of 3000 mm and a height of 6168 mm. The conveying throughput can reach 3–10 t/h with a pipeline diameter of 100 mm. The sensor is mounted on the straight pipe section above experimental silo A, as shown in Figure 5b. Polypropylene pellets are fed into the pipeline through a hopper, while a PLC-controlled roots blower adjusts the bypass ratio to regulate the conveying air flow and thus the particle velocity. The pilot plant is equipped with two experimental silos, enabling pellets to circulate between them. At the outlet, an offline weighing unit is installed to determine the average mass flow rate of the pellets.

## 4. Results and Discussion

### 4.1. Measurement Results from the Belt–Pulley Setup

**Figure 6:**
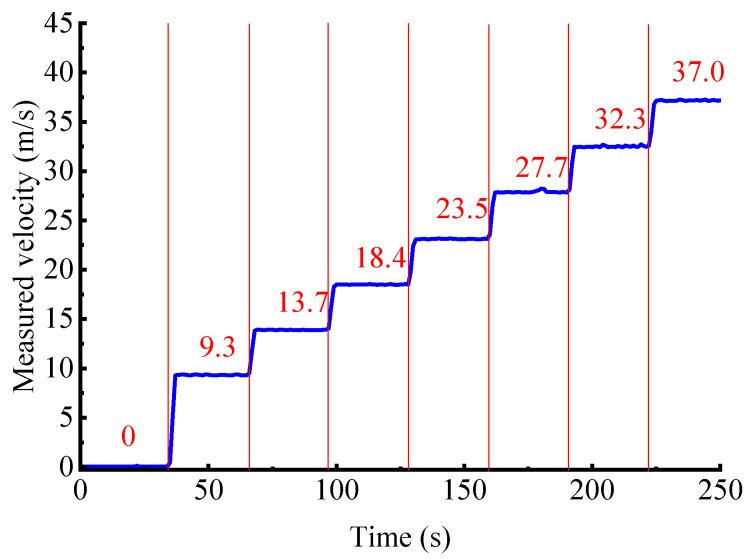
The measured velocity of the belt.

Figure 6 presents the measured velocity of the belt (*v*_c_) at seven motor speeds. It can be seen that *v*_c_ exhibits only minor fluctuation at each speed setting, with no abrupt jumps or drifts, indicating good measurement stability. Table 1 compares *v*_c_ with the actual velocity of the belt (*v*_r_). The maximum relative error between *v*_c_ and *v*_r_ does not exceed 1.82% across all test conditions, which is consistent with the approximate 1.87% velocity measurement uncertainty of the developed system, demonstrating the high accuracy of the measurement system for velocity measurement.

**Figure 7:**
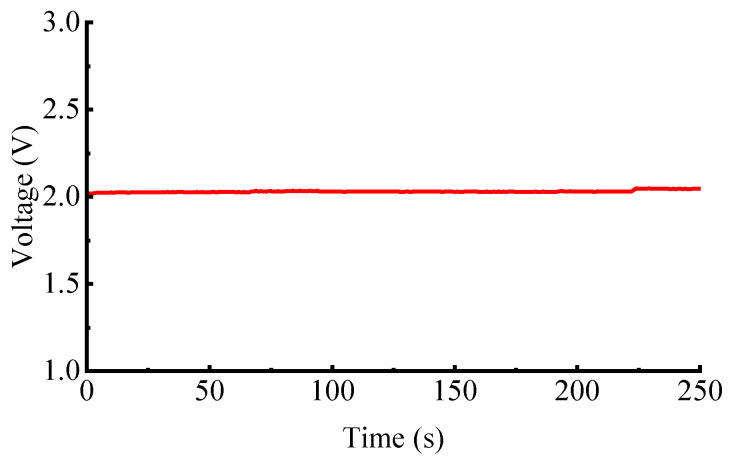
The capacitance signal.

Figure 7 shows the capacitance signal at seven motor speeds. Results show that the capacitance signal remains stable at approximately 2 V without noticeable drift, with a relative standard deviation of only 4.4%. Considering the fluctuation of the belt, the uncertainty of the capacitance measurement should be better, providing a reliable basis for subsequent concentration calibration.

### 4.2. Measurement Results from the Lab-Scale Pneumatic Conveying Rig

**Figure 8:**
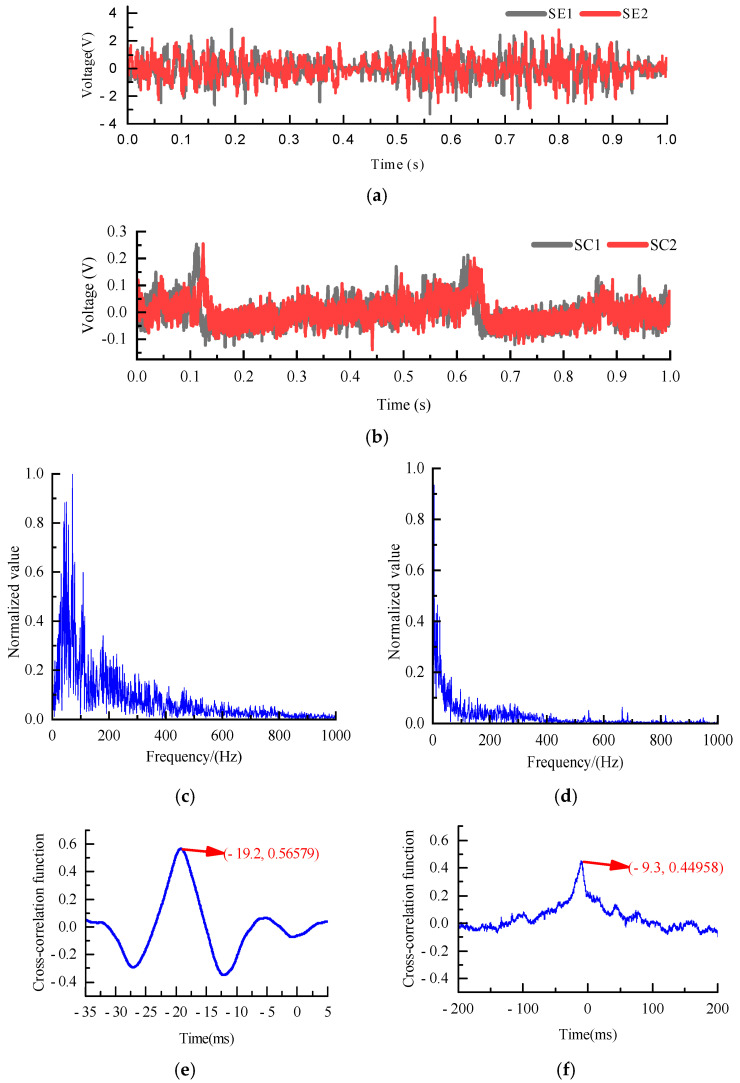
Electrostatic and capacitance signals, as well as their corresponding cross-correlation functions (45/20 Hz): (**a**) Electrostatic signals; (**b**) capacitance signals; (**c**) corresponding frequency spectrum of electrostatic signal SE1; (**d**) corresponding frequency spectrum of capacitance signal SC1; (**e**) cross-correlation function of electrostatic signals; (**f**) cross-correlation function of capacitance signals.

During the tests, the blower was operated at three frequencies (40 Hz, 45 Hz, and 50 Hz) in combination with various rotary valve frequencies between 15 Hz and 35 Hz, yielding ten distinct operating conditions. Figure 8a shows the electrostatic signals recorded by the upstream and downstream electrodes when the blower frequency was set to 45 Hz and the rotary valve frequency to 20 Hz. It can be seen that the two signals exhibited a pronounced similarity. Meanwhile, Figure 8b presents the original capacitance signals obtained simultaneously under the same operating condition, with the DC baseline removed. In comparison with the electrostatic signals, the capacitance signals are significantly weaker, primarily due to the relatively low volume concentration of polypropylene pellets in the pipe. Figure 8c,d show the corresponding frequency spectrums of electrostatic and capacitance signals. The signal frequencies are both in the order of 100 Hz, indicating that the sampling frequency of 20 kHz is enough. The cross-correlation function computed from the electrostatic signals is plotted in Figure 8e. The results reveal a distinct and well-defined peak. In contrast, the cross-correlation of the upstream and downstream capacitance signals (shown in Figure 8f) exhibits a peak amplitude markedly lower than that of the electrostatic signals, indicating a weaker correlation. It should be noted that the electrostatic signals have stronger correlations and more pronounced cross-correlation peaks than the capacitance signals, particularly under low-concentration conveying conditions. Using the electrostatic signal for cross-correlation yields more reliable and accurate velocity measurements for polypropylene pellets. Therefore, the electrostatic signal is preferred for velocity measurement. If the electrostatic signal is weak enough, the particle velocity can be calculated by cross-correlation of the capacitance signal.

**Figure 9:**
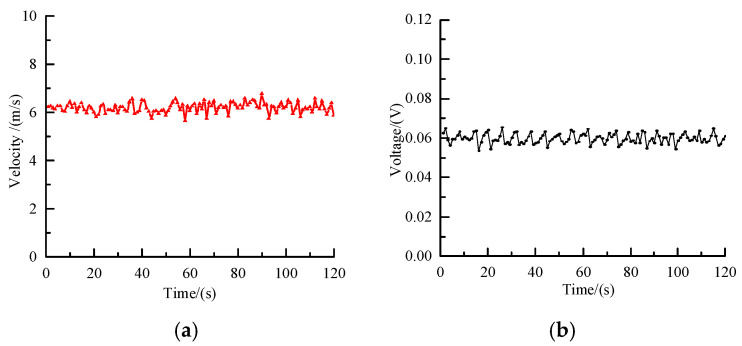
Measured velocity and capacitance signal (45/20 Hz). (**a**) Measured velocity; (**b**) changes in capacitance signal.

Figure 9a shows the measurements of polypropylene pellet velocities when the blower frequency was set to 45 Hz and the rotary valve frequency to 20 Hz. The results show that the velocities are mainly distributed in a range of 6–7 m/s. Small fluctuations caused by the periodic feeding of the rotary valve and flow-field disturbances inside the pipe are observed, but no pronounced flow instabilities occur during the conveying process. Figure 9b presents the corresponding capacitance signal under the same condition. The change of the capacitance signal remains stable at approximately 0.06 V, with minimal fluctuation, indicating only minor changes in polypropylene pellet concentration and a generally steady conveying state.

**Figure 10:**
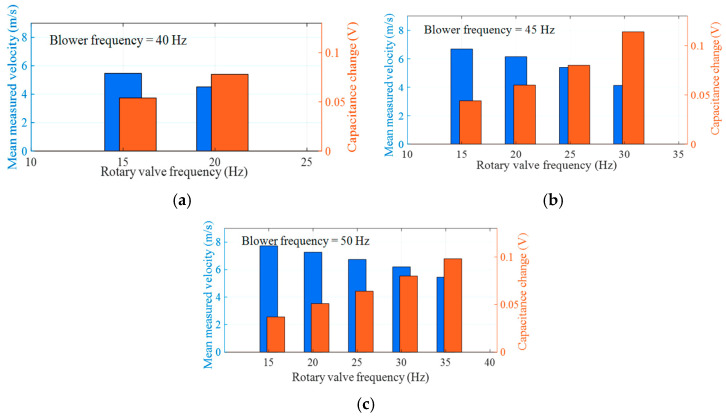
Mean measured velocities and changes in mean capacitance signal for all tested conditions: (**a**) Blower frequency = 40 Hz; (**b**) blower frequency = 45 Hz; (**c**) blower frequency = 50 Hz.

Figure 10 shows mean measured velocities and changes in mean capacitance signal for polypropylene pellets under all tested conditions. The results show that, under a fixed blower frequency, the measured velocity decreases while the amplitude of the capacitance signal increases with rising rotary valve frequency. This phenomenon arises from the increased particle feed rate, which leads to a higher particle concentration within the pipeline. These findings indicate that the measurement results are consistent with actual flow behavior, thereby providing a preliminary verification of the measurement feasibility.

A quantitative relationship between mass concentration and capacitance signal needs to be established. Table 2 presents the cumulative mass flow rate for conveyed pellets obtained by the weighing device. By combining Equation (4) with the mean velocity data from Table 2, the mean mass concentration can be calculated. Experimental data at the blower frequency of 45 Hz were employed as the calibration set. A second-order polynomial fit yielded an excellent correlation with a determination coefficient of 0.998, expressed as follows:

(5) $$\beta \;=\;168.46∆{V_{C}}^{2}\;+\;48.334∆V_{C}$$

where *β* is the mean mass concentration and ∆*V_C_* is the change in voltage of the capacitance signal caused by variations in concentration. Using the fitted relationship in Equation (5), the mass concentration can be calculated online from the capacitance signal.

**Figure 11:**
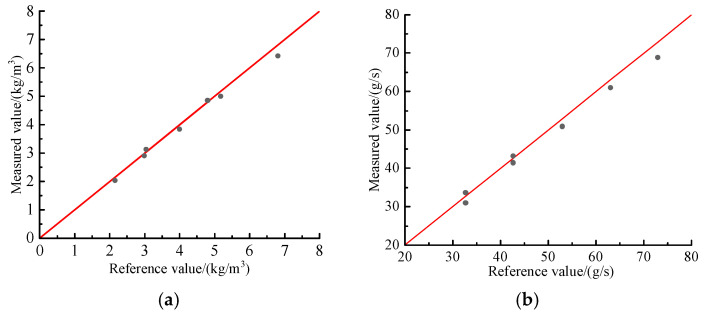
Comparison of mass concentrations and mass flow rates measured by the online measurement system with the reference values obtained by the weighing device: (**a**) Mass concentration; (**b**) mass flow rate.

To verify the accuracy of the relationship expressed in Equation (5), tests were conducted at blower frequencies of 40 Hz and 50 Hz. Figure 11 compares the mass concentrations and mass flow rates obtained from the online measurement system with the reference values calculated by the weighing device. The results indicate that the online measurements agree well with the reference values, with measured mass concentration ranging between 2.04 and 6.43 kg/m^3^ and a maximum relative error of approximately 5.5%. This error primarily arises from two factors. First, the relatively low mass concentrations during the tests result in small variations in the capacitance signal, making the measurements more susceptible to circuit instability. Second, inherent instabilities in the conveying system—such as nonuniform particle distribution and flow disturbances—reduce the stability of the capacitance signal, thereby introducing additional error. Overall, the measurement results demonstrate good accuracy and stability, confirming the feasibility and effectiveness of calibrating and determining mass concentrations and mass flow rates of polypropylene pellets based on the capacitance signal.

### 4.3. Measurement Results from the Pilot-Scale Pneumatic Conveying Rig

**Figure 12:**
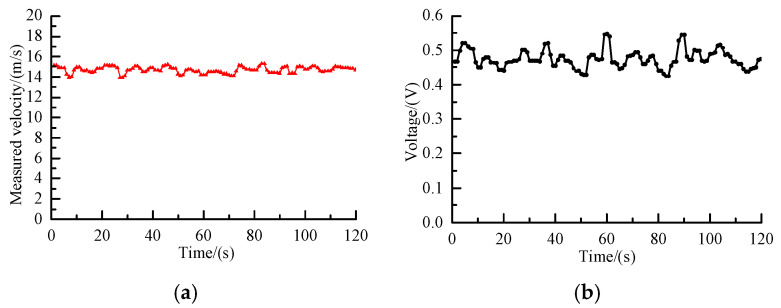
Continuous measurement results at a blower bypass ratio of 40%: (**a**) Measured velocity; (**b**) capacitance signal.

Six operating conditions were tested by setting the roots blower bypass ratio to 10%, 20%, 30%, 40%, 50%, and 60%. Figure 12a presents the measured velocity of polypropylene pellets at a blower bypass ratio of 40%. Although the velocity fluctuates within 14–16 m/s, it remains relatively stable; the observed fluctuations are mainly attributed to the pulsating feed rate and valve operation. In addition, the long pipeline with multiple bends further amplifies velocity oscillations.

The corresponding capacitance signal under the same condition is shown in Figure 12b. The signal fluctuates between 0.4 and 0.5 V, which can likewise be ascribed to pulsations of the feeder and valves as well as local mass concentration variations induced by pipeline bends. Under this operating condition, the average mass flow rate measured by the weighing device was 3.75 kg/s. Based on Equation (4) and the measured velocity, the average mass concentration was calculated.

Experimental data at bypass ratios of 20% and 50% were used for calibration. The functional relationship between the mean mass concentration (*β*) and the capacitance signal voltage variation (Δ*V_C_*) was subsequently derived, expressed as follows:

(6) $$\beta \;=\;-1.517{\Delta V_{C}}^{2}\;+\;57.415\Delta V_{C}$$

**Figure 13:**
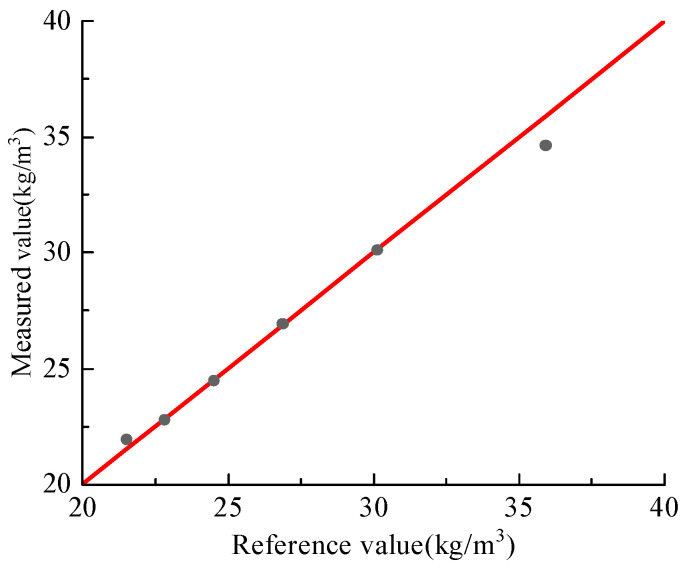
Comparison of mass concentrations measured by the online measurement system with reference values.

Equation (6) was further validated at bypass ratios of 10%, 30%, 40%, and 60%. As shown in Figure 13, the online mass concentrations obtained from the proposed measurement system agree well with the reference values measured by the weighing device across all test conditions, covering a mass concentration range of 30.98–68.87 kg/m^3^, with a maximum relative error of only 3.6%. These results demonstrate that the measurement system provides high measurement accuracy and stability in industrial particle transportation equipment.

## 5. Conclusions

This study developed an online measurement system for determining the mass flow of petrochemical particles in pneumatic conveying processes. The system demonstrated robust performance across different pipe diameters and particle concentration ranges in both lab-scale and pilot-scale test rigs. The velocity measurement uncertainty of the developed system was approximately 1.87%. In the lab-scale 50 mm pneumatic conveying, the system achieved mass flow measurement with a maximum relative error of only 5.5% for a particle concentration ranging from 2.04 kg/m^3^ to 6.43 kg/m^3^. In the pilot-scale 100 mm pneumatic conveying, the maximum relative error for the particle concentration measurement did not exceed 3.6% within a range of 30.98–68.87 kg/m^3^. These results indicate that the measurement system possesses strong adaptability and reliability in pneumatic conveying of petrochemical particles, highlighting its broad potential for engineering applications.

## Figures and Tables

**Table 1:** Comparison between measured and actual values of velocity.

Case	*r*_m_/r/min	*v*_r_/m/s	*v*_c_/m/s	Relative Error/%
1	590	9.3	9.2	1.08
2	891	14.0	13.7	1.23
3	1 192	18.4	18.5	0.54
4	1 493	23.4	23.5	0.42
5	1 794	28.2	27.7	1.77
6	2 096	32.9	32.3	1.82
7	2 368	37.2	37.0	0.54

**Table 2:** Mass flow rates obtained by the weighing device.

Rotary Valve Frequency/Hz	Mass Flow Rate/g/s
10	21.81
15	32.67
20	42.62
25	52.91
30	63.03
35	72.9

## Data Availability

Data supporting the findings of this study are available from the corresponding author upon reasonable request.
